# Department of Canine and Feline Medicine and Surgery

**Published:** 1900-05

**Authors:** Cecil French

**Affiliations:** Washington, D.C.


### DEPARTMENT OF CANINE AND FELINE MEDICINE AND SURGERY.

1. AN EXTRAORDINARY CASE OF HELMINTHIC MIGRATION.
2. HÆMATOMA OF THE SPLEEN.
3. NECROSIS OF THE ULNA, WITH FISTULA, IN A CAT.
4. NEOPLASM OF THE DUODENUM.

BY CECIL FRENCH, D.V.S.,
WASHINGTON, D.C.

1. AN EXTRAORDINARY CASE OF HELMINTHIC MIGRATION. During 1899, Miss Taylor, at that time assistant in the division of pathology of the Bureau of Animal Industry, had occasion to make a necropsy on the body of a mongrel puppy of medium size and seven or eight weeks old, which had died manifesting symptoms of peritonitis.

The whole intestinal tract was found to be swarming with round worms (ascaris canis). The liver might well be described as being in a similar condition, for the hepatic bile-ducts, gall-bladder, and intestinal bile-duct were seen to contain a multitude of these parasites. Some had perforated the surface of the liver, and, finding their way into the peritoneal cavity, had set up the peritonitis which had caused the animal's death.

The worms, when collected, almost filled a jar of about a half-litre capacity.

The above information was supplied to me by an official of the Department.

2. HÆMATOMA OF THE SPLEEN. Three occurrences of hæmatoma of the spleen have recently been discovered in the bodies of dogs dead of rabies, and examined at the pathological laboratory of the Bureau of Animal Industry. In one instance the tumor weighed some two pounds, and was composed of a well-organized blood-clot.

3. Necrosis of the Ulna, with Fistula, in a Cat. This animal had a fistulous sore of the left arm with several orifices of discharge, from which was emitted a purulent matter. Probing established the fact that the bony tissue was involved. The fluoroscope and skiagraph failed to reveal anything unusual excepting a slight opacity at one side of a spot on the outlines of the ulna.

An attempt was made for a few days to establish healthy granulations by injections of 4 per cent. solutions of formalin without any favorable results.

Ether was then administered and a tourniquet applied above the seat of lesion. The muscular tissue was incised and separated in the diseased area until the bone was freely exposed.

The neighboring periosteum was found to have undergone disintegration, and two very small orifices about half an inch apart were discovered on the shaft of the bone. The high serrations of a small amputating saw were employed to destroy the bone between the two orifices, so that a continuous open channel was established, and the point of a sharp curved bistoury to further pare away the diseased bone, the space being too small to permit of the use of the smallest curette on hand.

The wound was then thoroughly irrigated with formalin solution, and packed with xeroform gauze and left open. A moderately tight bandage was applied to the limb. The bandage was renewed daily, during which time there was no evidence of suppuration. At the end of four days the gauze was extracted, xeroform was freely powdered into the wound, and the edges were adjusted with silk sutures. In three weeks' time complete healing had taken place.

4. Neoplasm of the Duodenum. Some time ago I witnessed a tumor of the duodenum in a Russian wolf-hound. The animal was bred at the National Zoölogical Park, and later became the property of Mr. Charles Kramer, a wine merchant of this city. At the time of its death it was about three and a half years of age. It had been ailing some six weeks, and when seen by me the day previous to its death was in a condition of intense marasmus. The necropsy revealed the presence of a circumscribed, demarcated growth in the duodenum, about an inch and a quarter in length, and with an extreme thickness of not over half an inch, and the posterior border of which almost reached the ductus choledochus. The pylorus was not involved, neither was there any metastatic growth nor any other lesion elsewhere. The stomach was enormously dilated and much hypertrophied. The lumen of the canal at the point of lesion was completely obliterated and the animal had succumbed to starvation. I removed the affected portion of the gut and laid the same aside. The mucosa was intact, but the muscularis and serosa were replaced by a very firm, uneven, grayish, fibrous material, which in cross-section showed the presence of little, scattered, whitish bodies.

At this point I was called away, and on my return, much to my disappointment, I found the specimen to be no more, it having been confided to the drains through the gross stupidity of a negro attendant. I was thus deprived of all opportunity of determining its identity by microscopical examination. At that time I felt unwilling to record the case in such a necessarily incomplete manner, in spite of its great rarity, but I have since found an instance of carcinoma of the pylorus described by Eberlein, of Berlin, in *Monatshefte für praktische Thierheilkunde*, 1896 and 1897, p. 289, which has some points of resemblance to the tumor in question. Beyond this single instance I have been unable to find any other account of tumor occurring in this portion of the alimentary tract.

Müller, in his *Die Krankheiten des Hundes*, makes the bare statement that carcinoma of the stomach has been observed in postmortem examinations, but gives no references. In Eberlein's case the animal was a Leonberger, eight years old. Death was caused by cholæmia through closure of the orifice of the ductus choledochus, owing to an extension of the growth into the duodenum. The pylorus was the main seat of the tumor, which involved the circumference, with the exception of a line of about a finger's breadth, and had attained a thickness in places of nearly three-quarters of an inch. The pyloric opening was sufficiently large to permit of the passage of the little finger. In direct connection with this growth was another which was located at the region of the orifice of the ductus choledochus. Metastatic growths were also present in the liver and lungs.

The tumor was firm to the cut, and a grayish-white solid tissue had replaced the muscular and mucous coats. Cross-section revealed the presence of small, scattered, whitish or yellowish bodies of pin-head size. The carcinomatous nature was demonstrated by microscopical examination.

It becomes a matter of some difficulty to speculate on the identity of my tumor. The circumscribed character, judging from human conditions, would point toward carcinoma. The macroscopical appearance showed some resemblance to Eberlein's, but the age of the animal would seem not to favor this hypothesis, though Fröhner's statistics have shown that in sixty-five dogs treated by him for carcinoma in other regions of the body ten were affected between two and four years of age. The fact that the mucosa was intact adds to the difficulty. Carcinoma in man, when not originating as a primary lesion of the epithelial cells, is generally the result of extension of the growth from the pancreas, but in the case in question the latter organ was in normal condition, at least to the naked eye. The fact that the growth was of a very firm, schirrous character would hardly favor the hypothesis of sarcoma, but it is of course possible that it may have been a fibro-leiomyoma.

